# Development and Validation of a Risk Prediction Model for Foot Ulcers in Diabetic Patients

**DOI:** 10.1155/2023/1199885

**Published:** 2023-02-16

**Authors:** Jing Lv, Rao Li, Li Yuan, Feng-Mei Huang, Yi Wang, Ting He, Zi-Wei Ye

**Affiliations:** ^1^West China Hospital Endocrinology and Metabolism Department, West China School of Nursing, Sichuan University, Chengdu 610041, China; ^2^West China School of Nursing, West China Hospital Endocrinology and Metabolism Department, Sichuan University, Chengdu 610041, China

## Abstract

**Background:**

The current study analyzed the status and the factors of foot ulcers in diabetic patients and developed a nomogram and web calculator for the risk prediction model of diabetic foot ulcers.

**Methods:**

This was a prospective cohort study that used cluster sampling to enroll diabetic patients in the Department of Endocrinology and Metabolism in a tertiary hospital in Chengdu from July 2015 to February 2020. The risk factors for diabetic foot ulcers were obtained by logistic regression analysis. Nomogram and web calculator for the risk prediction model were constructed by R software.

**Results:**

The incidence of foot ulcers was 12.4% (302/2432). Logistic stepwise regression analysis showed that BMI (OR: 1.059; 95% CI 1.021-1.099), abnormal foot skin color (OR: 1.450; 95% CI 1.011-2.080), foot arterial pulse (OR: 1.488; 95% CI: 1.242-1.778), callus (OR: 2.924; 95%: CI 2.133-4.001), and history of ulcer (OR: 3.648; 95% CI: 2.133-5.191) were risk factors for foot ulcers. The nomogram and web calculator model were developed according to risk predictors. The performance of the model was tested, and the testing data were as follows: AUC (area under curve) of the primary cohort was 0.741 (95% CI: 0.7022-0.7799), and AUC of the validation cohort was 0.787 (95% CI: 0.7342-0.8407); the Brier score of the primary cohort was 0.098, and the Brier score of the validation cohort was 0.087.

**Conclusions:**

The incidence of diabetic foot ulcers was high, especially in diabetic patients with a history of foot ulcers. This study presented a nomogram and web calculator that incorporates BMI, abnormal foot skin color, foot arterial pulse, callus, and history of foot ulcers, which can be conveniently used to facilitate the individualized prediction of diabetic foot ulcers.

## 1. Introduction

Diabetic foot (DF) is one of the most serious complications in diabetic patients [1]. People with diabetes have 25% chance of lifetime risk of developing DF, with a global prevalence of 6.3% [2]. In China, the prevalence of foot ulcers among diabetes patients within a year was 8.1%, and it was 31.6% among patients who had a history of foot ulcers within a year [3]. The prevalence of diabetic foot ulcers (DFU) varies greatly in different countries and regions, ranging from 1.5% to 16.6% [2], with the characteristics of a high disability rate [1, 4], high mortality rate [5], high recurrence rate [3, 6–8], high medical expenses [9], long hospital stay [10], poor prognosis, and so on, which brings huge pressure to medical and healthcare.

Early screening of high-risk groups of diabetic foot ulcers and targeted strengthening of diabetic foot ulcer prevention and individualized interventions have important scientific and practical significance for the prognosis of diabetic patients. However, existing diabetic foot screening such as the diabetic foot risk screening system [11–15] and diabetic foot risk prediction model [16–22] are still in exploratory practice and have not been standardized. Therefore, based on the current status of diabetic foot prevention and management, this study intends to quantify the risk of foot ulcers in diabetic patients in the form of a nomogram and web calculator by constructing a risk prediction model, so as to provide a basis for further hierarchical management implementation.

## 2. Methods

### 2.1. Study Population

**Figure 1:**
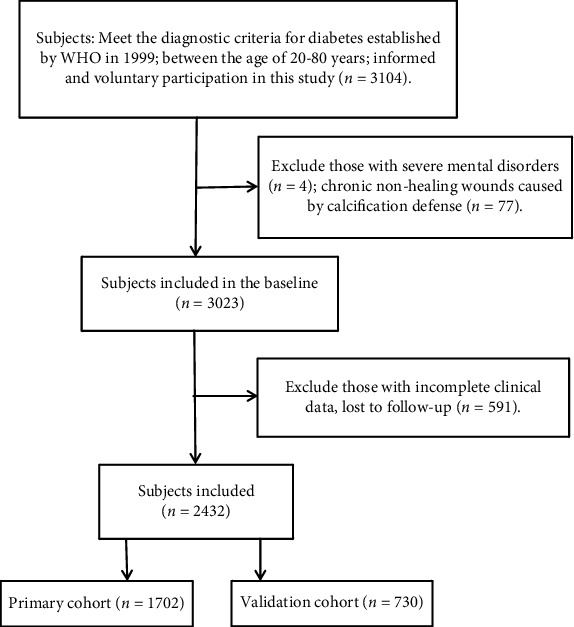
Flow chart of study participants.

This study was a prospective cohort study that used the method of cluster sampling. The outpatient and inpatient wards of the Department of Endocrinology and Metabolism of a tertiary hospital in Sichuan Province were included in the study from July 2015 to February 2020. Inclusion criteria are as follows: (1) meet the diagnostic criteria for diabetes established by WHO in 1999, (2) between the age of 20 and 80 years; and (3) informed and voluntary participation in this study. Exclusion criteria are as follows: (1) patients with severe mental disorders, (2) chronic nonhealing wounds caused by calcification defense, and (3) patients with incomplete clinical data and lost to follow-up. There were 2432 patients enrolled in total (Figure 1). The local ethics commission gave its approval to the current study.

### 2.2. Research Tools

#### 2.2.1. The General Information Questionnaire

The general information questionnaire includes (1) sociodemographic characteristics (gender, age, education level, marital status, body mass index, work status, income, medical insurance, living habits (smoking history, drinking history, and exercise status), etc.) and (2) disease information (diabetic peripheral neuropathy, diabetic retinopathy, diabetic nephropathy, diabetic lower extremity arterial disease, history of diabetic foot ulcer or amputation, duration of diabetes, and glycosylated hemoglobin).

#### 2.2.2. Diabetic Foot Risk Screening Assessment Form

The screening scale was made by simplifying the assessment scale for high-risk factors of diabetic foot. The overall Cronbach alpha value of the assessment scale for high-risk factors of diabetic foot was 0.920. The degree was 0.961 [23]. The simplified diabetic foot risk screening assessment form was assessed by 5 experts, with a total of 9 items, mainly including the patient's foot color change, foot shape change, abnormal toenails, protective sensation, foot arterial pulsation (decreased or disappearance), and skin conditions (callus, corns, fungal infection, and paronychia), the reliability of each item was 0.812-1.000, and the validity was 0.800-1.000, which has good reliability and validity.

### 2.3. Diagnostic Criteria and Related Definitions

This study defined the diabetic foot according to the Chinese Guidelines for the Diagnosis and Treatment of the Diabetic Foot. Diabetic foot refers to lower extremity infection and the formation of superficial or deep ulcers in diabetic patients due to neuropathy and lower extremity vascular disease [1]. In this study, diabetic foot ulcers were defined as diabetic foot patients with diabetic foot Wagner grade 1 and above. The occurrence of diabetic foot ulcers was considered a binary variable (0: no diabetic foot ulcers and 1: diabetic foot ulcers).

### 2.4. Data Collection

The demographic information and disease data of the research subjects were obtained through the electronic medical record database in the electronic information management system. Partial foot screening was performed by trained and qualified diabetes specialist nurses during the first basic foot condition screening after the subjects were enrolled. The outcome indicators of the research subjects were collected by telephone follow-up. A unified follow-up guidance questionnaire was used to evaluate the occurrence of foot ulcers and location one year after the subjects were enrolled.

### 2.5. Statistical Analysis

IBM SPSS Statistics 23.0 statistical software and R 4.1.2 software were used for statistical analysis and data processing. The R software was used to randomly divide the data into the primary cohort and the validation cohort at a ratio of 7 : 3 for random split validation.

#### 2.5.1. Statistical Description and Analysis

(1) Continuous variables
  - Normal distribution was represented by mean ± standard deviation, and *t*-test was used for statistical analysis
  - Nonnormally distributed data were expressed as median and quartile, and the rank sum test was used for statistical analysis
(2) Categorical variables: described by frequency and percentage and analyzed by chi-square test and rank sum test

In univariate analysis, variables with statistically significant differences (*P* < 0.1) were diagnosed as collinearity, and the predictive variables were screened by logistic multiple linear regression analysis, with screening criteria (*α*_in_ = 0.05 and *α*_out_ = 0.10). Use the “Irm” package and the “rms” package in the R software to develop the predictive model nomogram and web calculator. The performance of the model was evaluated using the indicators “Discrimination” and “Calibration.”

## 3. Results

### 3.1. Characteristics of the Study Population

A total of 2432 diabetes patients were recruited. There were 1293 males (53.2%) and 1139 females (46.8%); the age ranged from 20 to 80 years, with an average of 60.3 ± 13.9 years; 1791 (73.6%) were married; 2309 (94.9%) were Han nationality; 2199 cases (90.4%) patients had medical insurance; average BMI was 24.3 ± 3.9 kg/m^2^; disease duration was 1-48 years, with a median of 9 years; there were 897 cases (36.9%) of smoking; 784 cases (32.2%) of drinking; 4.6%-9.72% of glycated hemoglobin, with an average of 8.6 ± 2.3%; 429 cases (17.6%) of diabetic retinopathy; 89 cases (3.6%) of diabetic nephropathy; 515 cases (21.2%) of peripheral neuropathy; 398 cases (16.4%) of lower extremity vascular disease; 463 patients (19.0%) with a history of foot ulcers; and 52 patients (2.1%) with a history of amputation.

### 3.2. Patient Follow-Up Results

Among the 2432 patients who completed the follow-up, 302 diabetic patients developed foot ulcers, and the incidence of foot ulcers in diabetic patients within 1 year was 12.4% (302/2432). Among the 463 diabetic patients with a history of foot ulcers, 142 cases (30.7%) developed foot ulcers. Among the 1969 diabetic patients with no history of foot ulcers, 160 cases (8.1%) developed foot ulcers. Of the 302 patients with foot ulcers, there were 318 ulcers, 126 on the forefoot, 78 on the heel, 46 on the dorsum, 39 on the ankle, and 29 on other sites. Of these, 12 patients had 2 ulcers, 9 in the forefoot and dorsum, 2 in the forefoot and other areas, and 1 in the heel and ankle. Two patients had 3 ulcers, in the forefoot plus dorsum plus other sites and in the heel plus ankle plus dorsum.

### 3.3. Univariate Analysis and Logistic Regression Analysis of Diabetic Foot Ulcer

Univariate analysis showed that age, BMI, marriage, medical insurance, drinking, duration of diabetes, glycosylated hemoglobin, abnormal foot skin color, protective sensation, changes in foot shape, foot arterial pulse, callus, corns, abnormal toenails, paronychia, diabetic retinopathy, history of ulcer, history of amputation, and peripheral neuropathy were significantly different among the foot ulcers and no foot ulcers (*P* < 0.1).

Foot ulcers were used as the dependent variable, and those variables that were statistically significant in the univariate analysis were used as independent variables for stepwise regression analysis (*α*_in_ = 0.05 and *α*_out_ = 0.1). The results showed that BMI (OR: 1.059; 95% CI: 1.021-1.099), abnormal foot color (OR: 1.450; 95% CI: 1.011-2.080), foot arterial pulse (OR: 1.488; 95% CI: 1.242-1.778), callus (OR: 2.924; 95% CI: 2.133-4.001), and history of ulcer (OR: 3.648; 95% CI: 2.563-5.191) were risk factors of diabetic foot ulcers (Table 1).

### 3.4. Development of a Risk Prediction Model for Diabetic Foot Ulcers

According to the logistic regression analysis results, the formula of the risk prediction model for diabetic foot ulcers is constructed as follows:

(1) Z=−4.299+0.057 BMI+0.372 abnormal foot skin color+0.398 foot arterial pulse+1.073 callus+1.294 history of ulcer,P=11+e^−−4.299+0.057 BMI+0.372 abnormal foot skin color+0.398 foot arterial pulse+1.073 callus+1.294 history of ulcer.

**Figure 2:**
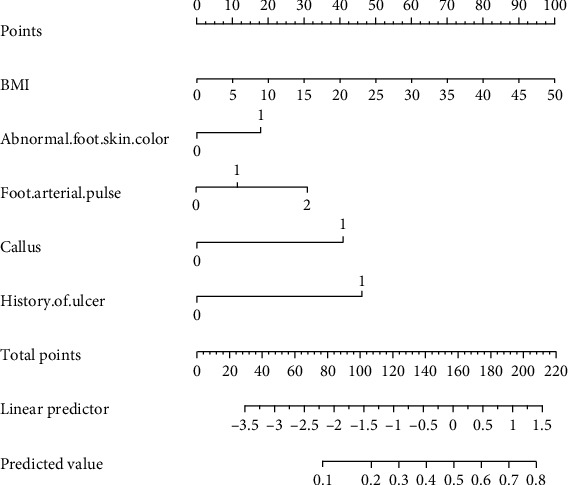
Nomogram of the risk prediction model for foot ulcers in diabetic patients.

**Figure 3:**
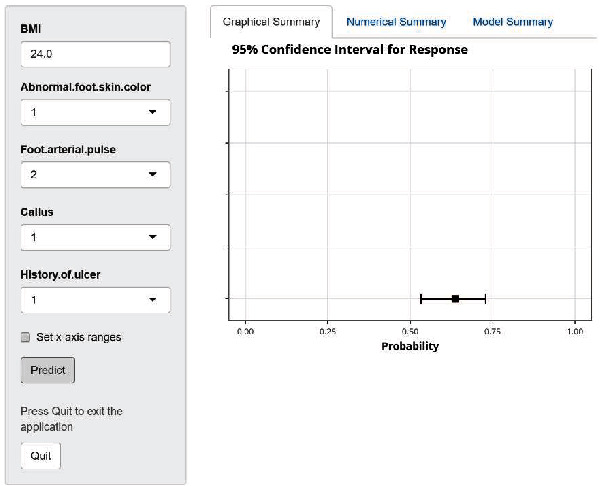
Web calculator for predicting foot ulcer risk in diabetic patients.

Based on the prediction model formula, the nomogram and web calculator of the diabetic foot ulcer risk prediction model constructed by R4.1.2 software are shown in Figures 2 and 3.

### 3.5. Diabetic Foot Ulcer Risk Prediction Model Internal Validation

#### 3.5.1. Distinguish Ability of the Model

**Figure 4:**
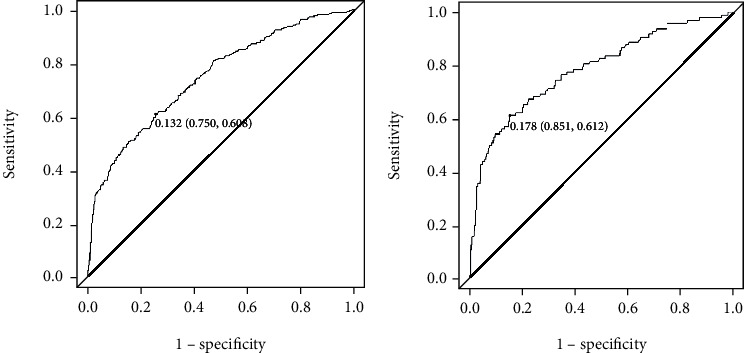
ROC curve of the risk prediction model for diabetic foot ulcer. (a) ROC curve of the diabetic foot ulcer risk prediction model in the primary cohort. (b) ROC curve of the diabetic foot ulcer risk prediction model in the validation cohort.

The ROC curve was used to evaluate the discriminative ability of the model, and the AUC value of the area under the ROC curve of the primary cohort was 0.741 (95% CI: 0.7022-0.7799), the sensitivity was 0.750, the specificity was 0.608, and the maximum value of the Youden index (0.358) was obtained. The best cutoff value was 0.132. The AUC value of the area under the ROC curve of the validation cohort was 0.787 (95% CI: 0.7342-0.8407), the sensitivity was 0.851, the specificity was 0.612, the maximum value of the Youden index was 0.463, and the optimal cutoff value was 0.178 (Figure 4).

#### 3.5.2. Calibration Ability of the Model

**Figure 5:**
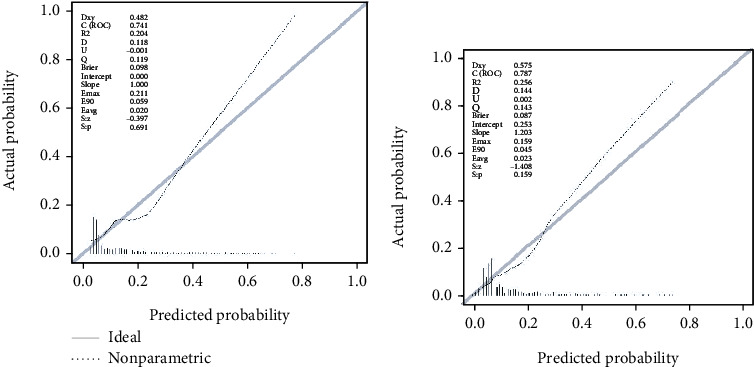
Calibration curve of the diabetic foot ulcer risk prediction model. (a) Calibration curve of the diabetic foot ulcer risk prediction model in the primary cohort. (b) Calibration curve of the diabetic foot ulcer risk prediction model in the validation cohort. The gray solid line “Ideal” at the zero-crossing point represents the ideal curve, and the black-dotted line “Nonparametric” represents the actual prediction accuracy of the model. The closer Nonparametric is to Ideal, the better the prediction accuracy of the model.

The Brier value was used to quantify the degree of calibration of the prediction model, and the Brier values of the prediction model were obtained as 0.098 in the primary cohort and 0.087 in the validation cohort, indicating that the model was well calibrated. Degree of the model was good. Draw the calibration curve of the diabetic foot ulcer risk prediction model (Figure 5).

## 4. Discussion

The results of this study showed that the 1-year incidence of foot ulcers in diabetic patients was 12.4%, among which the incidence of foot ulcers in diabetic patients with a history of foot ulcers was 30.7%. It was similar to the research on domestic (31.6%) [3] and abroad (30.6%) [6] studies. The incidence of foot ulcers in diabetic patients without a history of foot ulcers was 8.1%. It was same to the research of Jiang et al. (8.1%) [3], but higher than the results of the study by Zhou et al. (3.6%) [22]. This may be related to the different ways in which foot ulcers were collected in Zhou et al.'s study, which only included patients who voluntarily went to the hospital after foot ulcer, and may have overlooked some patients who developed an ulcer on the foot but did not seek prompt medical attention, resulting in a low detection rate of foot ulcers. In addition, this study found that the most common site of diabetic foot ulcers was the forefoot, followed by the heel.

The incidence of diabetic foot ulcers is high, and the clinical risk is great, and foot ulcers are a sign of the deteriorating health status of diabetic patients [24]. By developing a foot ulcer risk prediction model, early screening of people at high risk of diabetic foot ulcers and appropriate interventions are of great scientific and practical significance to reduce the risk of diabetic foot ulcers and improve patients' quality of life. In this study, we developed and validated a nomogram and web calculator model for the individual prediction of foot ulcers in diabetic patients. The regression analysis concluded that diabetic patients with increased BMI, abnormal foot skin color, abnormal foot arterial pulse, callus, and a history of ulcers were more likely to develop foot ulcers.

The findings of this study demonstrated a positive correlation between increased BMI and the occurrence of diabetic foot ulcers. Previous research revealed that obesity and an elevated BMI were both potential risk factors for diabetic foot ulcers [16]. Still, no studies have yet included BMI as a predictor in foot ulcer prediction models. Increased BMI can cause insulin resistance and pancreatic islet B cell malfunction [25]. Most obese patients have disorders of hepatic glucose metabolism and triphosphate cycle metabolism, leading to atherosclerosis in patients [26–29], causing a reduction in the blood supply to the patient's lower limbs, and if a local wound develops, the hypoxic environment will be detrimental to wound healing, which in turn contributes to the development of diabetic foot ulcers [30]. A simple physical examination can obtain this indicator to serve as one of the predictors of foot ulcers, which is a new factor in this study.

In this study, the callus, abnormal foot arterial pulse, and abnormal foot skin color were all significant local risk factors for diabetic foot ulcers. This was consistent with the results of previous studies that analyzed factors influencing diabetic foot [9, 28, 31–33]. The same factors as the predictive model for foot ulcers are constructed by Zhou et al. [22]. This study followed 477 diabetic patients and developed a predictive model for foot ulcer risk by logistic regression analysis, ultimately including duration of diabetes, nephropathy, retinopathy, history of ulcers, presence of skin changes, fungal of skin changes, callus or corn, fungal toenail, abnormal foot skin temperature, dorsalis pedis pulse diminution, and loss of protective sensation. The predictive model had a discrimination AUC = 0.77, no calibration of the model was reported, no internal or external validation was performed, and no visual model presentation was available.

It was also shown that diabetic patients with a history of foot ulcers were positively associated with the risk of foot ulcers (OR: 3.648), similar to Boyko et al. [16]. The presence of diabetic foot ulcers predicts a worse clinical course in diabetes patients and a higher risk of recurrent ulceration, amputation, and mortality as compared to those without such lesions [19].

Each of the currently available models for predicting the risk of the diabetic foot has its own characteristics. Some risk prediction models contain factors that require special tests to obtain, limiting the assessment's location and limiting its use in some primary care settings. In this study, the model was developed based on a large cohort study, and the predictors included can be measured directly. The nomogram prediction model is simple to use and allows the assessor in both clinical and primary care settings to derive the probability of foot ulcer occurrence for each patient from the nomogram, avoiding some of the limitations of some scoring systems, such as imprecise prediction results and cumbersome assessment methods. The assessment results also allow medical staff to accurately screen patients at risk of foot ulcers and implement targeted prevention strategies. The web calculator can be introduced directly into the healthcare system to quickly predict the incidence and risk score of diabetic foot ulcers after assessing risk factors in diabetic patients, providing a basis for clinical staff to make decisions on graded management. In addition, as the web calculator can be used online, it is more beneficial for future out-of-hospital dissemination of the model, providing an online prediction tool for designing and applying a diabetic foot ulcer care decision support system. We validated the model's discrimination by the area under the roc curve AUC value, the calibration of the model by the Brier value, and the optimal threshold value based on the Youden index, and considering sensitivity and specificity, the model performs well.

Our study also has some limitations. Firstly, the model in this study was constructed through baseline data collection and follow-up, which may be subject to selection bias due to missing data and lost visits reducing the adequate sample size. Secondly, as this study proposed to construct a universal foot ulcer risk prediction model without age stratification of the included population, the screening of model predictors may be subject to bias. Finally, further multicenter external validation should be performed in future studies so as to improve the stability and generalizability of the model.

## 5. Conclusion

In this study, the investigation and follow-up of diabetic patients in a tertiary hospital in Chengdu, Sichuan Province, found that the incidence of foot ulcers in diabetic patients within 1 year was 12.4% and the most common site of occurrence was the forefoot. BMI, abnormal foot color, abnormal arterial pulse, callus, and history of foot ulcers were risk factors for foot ulcers in diabetic patients. The prediction model of foot ulcer risk was constructed by the regression results. The obtained model has good prediction performance. The model was presented in a nomogram and web calculator, which was convenient for clinical use. The constructed risk prediction model has the characteristics of noninvasiveness, safety, convenience, economy, good repeatability, good patient compliance, and good clinical practical operation and has extremely high promotion value in clinical and basic medical institutions.

## Figures and Tables

**Table 1:** Multivariate logistic regression analysis of diabetic foot ulcers.

Variable		*β*	SE	*Z*	*P*	OR (95% CI)
BMI		0.057	0.090	3.048	0.002	1.059 (1.021-1.099)

Abnormal foot skin color	No Yes	0.372	0.185	2.006	0.040	1.450 (1.011-2.080)

Foot arterial pulse	Normal Weaken Disappear	0.398	0.091	4.356	<0.001	1.488 (1.242-1.778)

Callus	No Yes	1.073	0.160	6.694	<0.001	2.924 (2.133-4.001)

History of ulcer	No Yes	1.294	0.180	7.195	<0.001	3.648 (2.563-5.191)

Constant		-4.299	0.496	-8.665	<0.001	0.013

## Data Availability

The reader can access the data by correspondence with the authors.
